# Digitization of myth: The HimmapanVR Project's role in cultural preservation

**DOI:** 10.1016/j.heliyon.2024.e30052

**Published:** 2024-04-20

**Authors:** Suepphong Chernbumroong, Perasuk Worragin, Natchaya Wongwan, Kannikar Intawong, Pipitton Homla, Kitti Puritat

**Affiliations:** a College of Arts, Media and Technology, Chiang Mai University, Chiang Mai, Thailand; b Faculty of Public Health Chiang Mai University, Chiang Mai, Thailand; c Department of Library and Information Science, Faculty of Humanities, Chiang Mai University, Chiang Mai, Thailand

**Keywords:** Visual museum, Digital museum, Cultural preservation, Cultural heritage, Immersive systems, Virtual reality, Mythical creatures

## Abstract

This study investigates the visitor experience at the '3D HimmapanVR' virtual museum, which focuses on the preservation and dissemination of cultural heritage related to Thai mythical entities, specifically the Himmapan animals. Despite their cultural significance, information about these creatures is limited and fragmented. The HimmapanVR initiative aims to mitigate this issue by establishing a virtual museum that curates and exhibits these entities via a virtual reality (VR) platform, thus enhancing their accessibility, educational value, and conservation. The project entails the digitization of artwork, the creation of 3D models of archaeological artifacts, and the utilization of digital paintings and animations to depict the three primary and fifteen subsidiary types of Himmapan creatures. The hypothesis posits that the virtual museum will influence users' Effort Expectancy (EE), Performance Expectancy (PE), and the perceived authenticity of the archaeological objects. Data collected from 30 participants indicate that the virtual museum effectively augments knowledge and engenders a sense of authenticity. However, enhancing the VR user experience remains a challenge. Conclusively, this study presents the inaugural virtual museum dedicated to Himmapan animals, which occupy an essential niche in Thai cultural heritage. To date, no existing physical or virtual museum offers an exhaustive compilation and presentation of various Himmapan creatures, a gap this project endeavors to fill.

## 1 Introduction

Mythical creatures such as the sphinx, kraken, dragon, and Cerberus have achieved global recognition. In Thai culture, there exists a diverse array of fascinating creatures, ranging from a majestic elephant adorned with six tusks to a colossal avian entity capable of parting the sea through the mere movement of its wings. These mythical beings, referred to as Himmapan creatures, inhabit the Himmapan Forest, an enchanted and distant realm abundant with mysteries and wonders. Himmapan creatures are creatures with strange and extraordinary characteristics that exist in the realm of imagination, and no one has ever seen them in reality. They are only known through ancient stories, and artists often create images of them based on their interpretations of these stories and their own imaginations. In Thailand, Thai mythical animals are influenced by beliefs and cultural narratives from other ethnic groups, which are blended with the Thai culture, resulting in the creation of unique Thai mythical animals. Himmapan animals are considered an important heritage that has been passed down through Thai generations. They are created through imagination and brought to life through the intricate and beautiful artistry. However, in today's world, knowledge about these animals is relatively scarce. Only those who are truly dedicated to studying and those with a genuine interest in these creatures have access to this information, and their numbers are not significant. Furthermore, information about these creatures is scattered and challenging to find. Most people are familiar with only a few famous mythical animals such as the Naga, Garuda, Singha, Erawan Elephant, Genuak, Kinaree, and Hongs. In reality, there are many more diverse species of these creatures that the general public may not be aware of. Currently, cultural heritage, including knowledge of mythical animals, is slowly fading away. Information about mythical animals is primarily confined to a narrow circle of artisans and artists, and the presentation is often limited to drawings and illustrations in various publications. There is a need for more engaging and accessible ways to present this knowledge to the general public for those interested in studying this subject [1]. As technology advances, virtual museums have emerged as a powerful tool to safeguard and share cultural heritage. Virtual museums can be described as museums without physical boundaries, allowing visitors to access them at their convenience [2].

Since Himmapan creatures are imagined creatures, showcasing them in physical museums poses challenges. Virtual museums, therefore, offer a more convenient avenue for their presentation. To overcome the limitations of traditional preservation methods and to improve global access to and engagement with intangible cultural heritage, VR has been chosen as the medium for preserving the cultural heritage of Himmapan animals. To illustrate, VR offers an immersive experience, allowing users to explore the Himmapan creatures and their mythological world in a way that traditional media cannot. This immersive aspect can significantly enhance engagement and learning outcomes, making the cultural heritage more accessible and appealing to a broader audience. Moreover, VR makes the inaccessible accessible. The Himmapan creatures, rooted deeply in Thai mythology, are complex, and their stories are not widely known outside certain scholarly circles or communities. Through VR, individuals around the world can access this cultural heritage without the physical and geographical limitations of visiting a museum in Thailand. Furthermore, VR enables for the digital preservation of cultural heritage in a way that is resistant to the decay and degradation that physical artifacts undergo. This is especially important for intangible cultural heritage, such as the stories about Himmapan creatures, which do not have a physical form to begin with. To our knowledge, no one has ever compiled information and images of Himmapan animals in a virtual museum. Therefore, the researchers intend to systematically gather information on mythical creatures known as Himmapan animals and present them in a virtual museum of Himmapan animals, as well as investigate the effects of implementing a virtual museum of Himmapan animals on users' expectations and experiences.

## 2 Literature review

### 2.1 Himmapan creatures

**Fig. 1:**
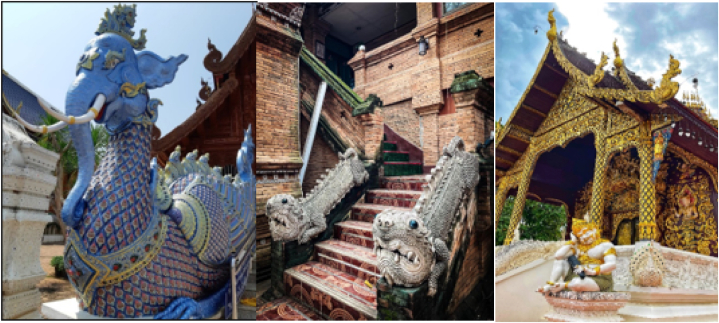
A Himmapan Animal in the Thai architectural culture of Wat Rong Suea Ten.

In the concept of the three worlds or Traibhumi, people believe that the Himmapan forest is located at the foothills of the Sumi Mountain, beneath Buddhist heaven. A variety of magic animals and Himmapan wildlife, reside there. The Himmapan wildlife are extraordinary creatures that originate from the realm of imagination. These animals are prevalent in diverse Thai literary works and artistic creations. To illustrate, Himmapan creatures are animals in literature or imagined creatures that have unique physical characteristics, different from typical animals. They are a blend of different species, created through the creative minds of artists in ancient times. These Himmapan creatures are recorded in the scriptures, specifically in the Tripitaka or Traibhumi, which mention the Himmapan forest as the habitat of these creatures. Stories about Himmapan creatures often come from ancient folklore and are narrated in various scriptures, and the interpretations may vary as no one has actually seen these creatures in reality [3]. Thailand's mythical animals are heavily influenced by beliefs and cultures, incorporating stories from various other ethnic groups. These elements are integrated into Thai culture, giving rise to uniquely Thai mythical animals. Thai culture has been significantly influenced by various sources, primarily from China, India, and Cambodia. For example, from India, Thai culture has adopted and adapted creatures like the Garuda, Naga, and many others, which are considered the ancestors of Thai mythical animals [3]. Evidence related to Himmapan animals during the Sukhothai period is found in the form of stories associated with Buddhist scriptures known as the "Traiphum." Additionally, there is evidence of the presence of mythical animals in ancient art and murals at various historical sites, although their numbers are relatively limited. As time progressed into the Ayutthaya period, additional evidence emerged in the form of decorative patterns, specifically found in cabinets containing Buddhist scriptures and religious texts. These patterns included depictions of Himmapan creatures and were used to store the Tripitaka and various Buddhist scriptures. Furthermore, these creatures appeared in royal ceremonies, especially during cremation ceremonies, where the number of mythical animal sculptures increased compared to the Sukhothai period. During the Rattanakosin period, it was observed that there was an increase in the creation of sculptures and depictions of mythical animals compared to earlier periods. This expansion was influenced by the growing imagination of individual artists and resulted in a wide variety of representations. These mythical creatures have been incorporated into various forms of presentation, such as cinema and government emblems, demonstrating the wide range of artistic expressions related to Himmapan animals. Fig. 1 depicts Elephant-based, Singha Mom (a four-legged creature), and Naga-based creatures on the rooftop of the Himmapan Animal exhibit, showcasing Thai architectural culture at Wat Rong Suea Ten. The main type and sub-type of Himmapan Animal are shown in Table 1.

**Table 1:** Main type and sub-type of Himmapan Animal.

Main types creatures	No	Sub-types Creatures	Description
Fish and Amphibian-based	1	Fish based	Graceful aquatic forms, often carved into temple and castle structures, symbolizing reverence for water sources and aquatic life in Thai culture.
	2	Crab Based	Recognized by their hard shells and formidable claws, these creatures are depicted in decorative motifs on temple entrances and castle gateways, symbolizing protection and tenacity.
	3	Naga Based	With serpentine bodies and jewel-like scales, Naga-based creatures are revered protectors found coiled along Buddhist temple staircases and architectural decoration and ornaments [24].
two-legged	4	Monkey based Creatures	Playful and agile, often depicted with long tails, they embody the lively spirit of Thai culture and can be seen in temple murals, castle reliefs, and sculptures.
	5	Bird based	Bird-based creatures, known for their graceful wings and beaks, adorn temple rooftops and feature prominently in Thailand's art, crafts, and official documents. They hold significant cultural value and serve as important symbols in South-East Asian arts and architecture [25].
	6	Human Based	Possessing supernatural attributes, they grace temple murals, castle frescoes, and sculpted statues, enriching Thai cultural heritage with compelling stories.
four-legged	7	Ghilen based	Towering giants that stand as sentinels at temple entrances and castle gates, symbolizing protection and spirituality.
	8	Deer based	Graceful and often adorned with antlers, they symbolize gentleness and a deep reverence for nature, found in temple interiors, castle embellishments, and sculptures.
	9	Lion based	Majestic and regal, they serve as guardian statues at temple gates, adorn castle entrances, and are sculpted into reliefs that exude bravery and nobility.
	10	Horse based	Elegantly sculpted, they capture equine beauty, gracing temple murals, enhancing castle decorations, and taking the form of exquisite sculptures.
	11	Rhino based	Possessing armored bodies and prominent horns, they symbolize resilience and fortitude, featured in temple and castle reliefs and intricately carved sculptures.
	12	Elephant based	Adorned with intricate designs, they pay homage to revered Thai elephants, symbolizing wisdom and strength, found in temple and castle architecture and ornate sculptures.
	13	Cattle based	Inspired by cows and bulls, they symbolize the prosperity of Thai agriculture, commonly found in temple motifs, castle embellishments, and intricately carved sculptures.
	14	Canine based	Resembling loyal and guardian canines, they evoke protection and trust, depicted in temple murals, castle motifs, and sculpted reliefs.
	15	Crocodile based	Inspired by the distinctive features of crocodiles, they represent adaptability and survival, often found in temple designs, castle architectural elements, and finely crafted sculptures.

### 2.2 Virtual museum

Researchers have proposed various definitions for virtual museums. According to McKenzie, the virtual museum is described as an online platform that utilizes digital technology to convert its collections into a digital format, eliminating the requirement for a physical location [4]. Schwaben provides a definition of virtual museums as institutions that house digitized artifacts and associated information, prepared through various multimedia technologies, and accessible globally via online platforms, eliminating the requirement for physical space [2].

Regarding the categorization of virtual museums, they were sorted into three main divisions based on the design of the virtual museum. These categories include the type of artwork, the method of transitioning into the virtual environment, and the approach to presenting information [2,4]. The first component of this classification, referred to as "type of artwork," involves the categorization of artworks within virtual museums into 8 distinct groups, including Culture-Art, Composite, Archaeology, Science/Technology, History, Military, Industry and Natural History. To explain, Culture-Art includes art galleries, art museums, and museums that house cultural objects and artifacts, while Composite museums offer a diverse collection of artifacts, incorporating archaeological findings from the region and ethnographic items portraying the lifestyles of local inhabitants. Archaeology museums exhibit artifacts unearthed through regional archaeological excavations, providing insights into the historical and archaeological heritage. In contrast, Science/Technology museums are focused on the fields of science and technology, aiming to guide visitors toward scientific exploration. History museums celebrate historical events and notable figures within the region, while Military museums document the social, historical, and technological evolution of military forces. Industry museums showcase the development of specific regional products or industries, and Natural History museums are focused on nature and the preservation of natural life in parks. Another criterion pertains to the transfer of virtual museums into the virtual environment, which can be categorized into 3 distinct modes, including real, semi-real, and fictional. To elaborate, a real virtual museum constitutes a comprehensive digital recreation of a real museum, replicating all of its components within the virtual realm. Conversely, a semi-real virtual museum is created by relocating a part of a physically existing museum into the virtual world or by assembling works from various museums under common themes. Lastly, a fictional virtual museum represents a unique type of virtual museum that exists solely within the virtual environment as a product of pure imagination, with no real-world counterpart. The last aspect to consider is the type of information presentation. In the 2D/3D Object category, virtual museums only visually present the artworks to their audience as either 2D or 3D objects with no information. Alternatively, in Informative Pop-Up virtual museums, information is provided to visitors through pop-up displays alongside visual representations of the artworks. On the other hand, Link/URL virtual museums not only visually exhibit the artworks but also direct visitors to web pages, allowing them to access further information about the artworks through embedded hyperlinks.

Recently, there has been a noticeable increase in the use of virtual museums. In the field of underwater cultural heritage, one example of a virtual museum called UnderwaterMalta, which uses traditional photography, video, 3D models, and virtual reality to present the underwater cultural heritage sites of the Maltese Islands to make these sites accessible to the public and to raise awareness of their importance. As a result, it is suggested that the virtual museum has the potential to attract a global audience and promote the conservation of underwater cultural heritage sites [5]. Marín-Morales et al. recreated the Institut Valencià d'Art Modern (IVAM) museum in a virtual 3D environment to test environmental simulations with head-mounted displays. They compared the experience of exploring art in a physical museum with a simulated version. They used a highly realistic 3D copy of the exhibition created in Rhinoceros v5.0, incorporating some real textures. The findings indicated significant time-dependent differences in navigation during the first 2 min of the virtual tours, but no differences in navigation behavior between physical and virtual museums thereafter [6]. The National Museum of Indonesia, responsible for safeguarding the cultural heritage of the Indonesian population, introduced an online virtual tour in response to the challenges posed by the COVID-19 pandemic. They employed a 360° panoramic image approach to offer detailed and extensive information and education to the public. This virtual tour incorporates both written and spoken information features. In summary, a survey revealed that 85.1 % of respondents found the information presented on this tour to be highly understandable [7]. An additional example is the Virtual Collections, which functions as a digital museum that presents cultural heritage artifacts from Argentina. It consolidates collections and resources from diverse regional institutions, creating a cohesive virtual exhibition. Researchers propose that virtual experimentation provides museums with a rapid and flexible means to design exhibitions. Moreover, these virtual structures can also be utilized to create interactive tours documenting past exhibitions and aiding in the prototyping of real-life exhibitions [8]. Additional virtual museums will be presented in the table below.

In this research, our virtual museum can be categorized as a fictional virtual museum specializing in the exhibition of culture-art-themed artworks, presented using 3D objects and informative pop-up features shown in Table 2. To provide more context, it's important to recognize that Himmapan animals hold a significant place in Thai cultural heritage and are considered a form of Thai art. In Thailand, there are only 2 physical museums, namely the Erawan Museum and the Garuda Museum, that showcase Himmapan animals. Nevertheless, there is no museum, either physical or virtual, that consolidates information on various other Himmapan creatures and presents them comprehensively. Hence, this virtual museum serves as a virtual environment featuring imaginative 3D objects that do not exist in reality. Furthermore, it offers visitors concise information about the artworks they explore through interactive pop-up descriptions, allowing them to gain insights into the objects while examining the artwork.

**Table 2:** Comparison of virtual museums.

Name of Virtual museum	Country	Artwork	Type of artwork	Type of transfer to virtual environment	Type of information presentation
Underwater Malta [5]	Malta	Shipwrecks	Archaeology	Real virtual museum	3D Object and Link/URL
The Institut Valencià d'Art Modern (IVAM) [6]	Spain	Sculptural works and video installations	Culture-Art	Real virtual museum	3D Object
National Museum of Indonesia [7]	Indonesia	Archaeology and ethnography collection	Composite	Real virtual museum	2D visual of the artefact and Informative Pop-Up
Virtual Collections [8]	Argentina	Cultural heritage objects	Culture-Art	Semi-real virtual museum	3D Object and Informative Pop-Up
Ancient Afrasiyab [9]	Uzbekistan	Archaeological artifacts	Composite	Semi-real virtual museum	3D Object and Informative Pop-Up
The Pleistocene Crete museum exhibition [10]	Greece	Life-sized reconstructions of Pleistocene Cretan fauna	Natural History	Semi-real virtual museum	3D Object and Informative Pop-Up
The Virtual Museum of Baghdad [11]	Iraq	Artifacts related to the cultural heritage of Iraq	Culture-Art	Semi-real virtual museum	3D Object, Link/URL and Informative Pop-Up
The virtual tour of the Civic Art Gallery of Ancona [12]	Italy	Ancient artworks	Culture-Art	Real virtual museum	2D visual of the artefact and Informative Pop-Up
Online virtual tour of the Exhibition of Architecture of the Forbidden City [13]	China	Cultural relics and historical materials	Culture-Art	Real virtual museum	2D visual of the artefact and Informative Pop-Up
A virtual tour of the Van Gogh Museum in Amsterdam [14]	Netherlands	Paintings by Vincent Van	Culture-Art	Real virtual museum	2D visual of the artefact
		Gogh and several other artists			
Anne Frank House [15]	Netherlands	An exhibition on the life and times of Anne Frank	History	Real virtual museum	2D visual of the artefact and Informative Pop-Up

Based on the comprehensive analysis of various virtual museums, to improve the HimmapanVR experience, the Visual Museum of Himmapan Animals should implement advanced VR technologies to offer visitors a more interactive experience. For example, incorporating motion controllers that allow users to interact with exhibits in a way that simulates real-world engagement can enhance immersion and educational value. In addition, providing detailed information about each exhibit through pop-up descriptions can significantly augment the learning experience. Moreover, emphasizing the use of high-quality 3D models to represent the Himmapan animals, ensuring they are rendered with attention to detail and accuracy, is crucial. By focusing on these functions, the HimmapanVR project can enhance its offering, ensuring it not only preserves but also enriches the cultural heritage of Thailand through an engaging and educational virtual museum experience.

## 3 The HimmapanVR project

The HimmapanVR project is an online platform that compiles a wide variety of mythical creatures from the Himmapan animal realm, gathering information about Himmapan creatures sourced from the "Thai Dum book", various artworks displayed at the Roitawarabarn Baandhawalai Museum and digital paintings and animations. Additionally, the HimmapanVR project has the potential to provide numerous advantages. It not only serves as a valuable resource for preserving and sharing cultural heritage but also has the potential to drive educational, research, and artistic endeavors while promoting tourism and cultural exchange.

### 3.1 The purpose of HimmapanVR and objective

*3D HimmapanVR:* The objective of the application of fully 3D virtual reality is to offer an immersive experience using specialized Head-Mounted Displays (HMDs) for visitors, providing them with an interactive digital preservation experience of Himmapan creatures. This involves the fully 3D reconstruction of objects from the "Thai Dum book", artworks displayed at the Roitawarabarn Baandhawalai Museum and digital paintings and animations, enabling meaningful engagement with these objects.

*Web-based digital collection of Himmapan creatures:* the objective of this system is to provide an educational experience tailored for students, librarians, archaeologists, and historians that simplifies access to information about Himmapan creatures. The system offers organized access to a repository containing comprehensive details on Himmapan creatures from the "Thai Dum book", artworks exhibited at the Roitawarabarn Baandhawalai Museum and digital paintings and animations. It also provides knowledge-based digitization services using the standard metadata format of VRA CORE.

### 3.2 The benefit and the content of the HimmapanVR

In this project, we conducted data collection on Himmapan animals sourced from the Thai Dum books from the reign of King Rama III. These books are considered ancient manuscripts, functioning as cultural treasures with immeasurable value, rarity, and no apparent contemporary reproduction to support ongoing documentation. They are highly esteemed as invaluable historical documents, reflecting the creative expressions of ancient Thai culture and artistry contained within each volume. Additionally, we gathered digital paintings and animations via social media campaigns, inviting artists to contribute their interpretations of Himmapan animals. This sought to showcase the evolving artistic imagination surrounding Himmapan creatures in contemporary times. Furthermore, we collected artworks displayed at the Roitawarabarn Baandhawalai Museum, an art museum and religious shrine that includes archaeological artifacts and wall sculptures depicting mythological creatures. The museum is located in the Suthep district of Chiang Mai, near the base of Doi Suthep mountain. However, the Thai Dum books are historical documents that are not readily accessible to the general public. Furthermore, knowledge about Himmapan animals remains relatively limited and is confined to those with a particular interest in mythological creatures. Additionally, the Roitawarabarn Baandhawalai Museum's location in Chiang Mai may pose travel inconveniences for those keen on visiting. To address these challenges and limitations, we utilized the concept of virtual reality, employing digital cultural systems to explore the Himmapan Animals Museum. This involves the fusion of virtual reality with visual technology to create immersive virtual objects for users. The use of virtual reality will offer a crucial solution, particularly for museums situated in less accessible areas. An overview of the project's content and its advantages within HimmapanVR will be described below.

- To offer users an engaging experience where they can virtually step into the archaeological world of Himmapan animal art, gaining a fresh perspective and delving into the knowledge about these creatures.
- In terms of education, the application serves as a learning resource for virtual-based learning. It offers comprehensive insights into the digital preservation of Himmapan creatures, incorporating standardized metadata. This resource is designed to be useful for students, librarians, and historians interested in Himmapan creatures.
- To overcome the constraints of physical museums, which often limit the number of visitors at a given time, the virtual reality platform offers unrestricted access for multiple users to explore exhibitions digitally. This means there are no limitations on the number of visitors or the duration of visits, ensuring everyone can enjoy the museum experience.

## 4 Visual museum design and development process

In this section, we will describe the visual museum design and development process, which encompasses the creation of the digital structure, digital archival objects, and the development of the visual museum for the virtual representation of Himmapan animals. An overview of the development process is illustrated in Fig. 9.

### 4.1 Visual museum and immersive design

The decision to develop the virtual museum in a specific format was driven by the aim to create an immersive storytelling experience centered on the myth of the Himmapan animals, thereby facilitating user engagement and learning. The virtual museum's structured layout supports thematic storytelling and logical progression of exhibits, enriching the narrative related to Himmapan cultural heritage. By incorporating interactive elements and multimedia resources within this format, we enhance learning opportunities, catering to diverse user preferences and reinforcing the museum's educational objectives. This design choice is pivotal to our research, allowing us to explore the effectiveness of virtual reality in the dissemination of culture and education. It ensures that the museum is accessible and provides a rich, engaging user experience for those interested in exploring Thai mythical creatures.

The Museum of HimmapanVR application utilizes Meta Quest 2 HMDs (Head-mounted Displays) to implement an exhibit system designed for visual museums. This virtual reality experience immerses visitors in scenes and objects that closely resemble reality, creating the sensation of being in a genuine museum dedicated to the Creatures of Myth from Himmapan animals within the visual museum setting. With a focus on immersive design, HimmapanVR seeks to introduce novel paradigms of interaction and immersion. This is achieved through the digitization of 3D scanned objects, encompassing the complete collection of sculptural heritage associated with Himmapan animals. Additionally, visitors have the opportunity to closely examine these objects and explore them from various angles using motion controllers. This interactive feature allows visitors to feel as though they are holding and interacting with real objects within the museum environment. HimmapanVR's immersive design not only enhances the visitor experience but also contributes to the preservation and exploration of cultural heritage related to Himmapan animals.

#### 4.1.1 Apparatus

The Meta Quest 2 was selected as the foundation for developing the visual museum. Our system is designed to be compatible with various virtual reality Head-Mounted Displays (HMDs), including Quest 1, HTC Vive Pro 2, and Valve Index VR, ensuring accessibility and flexibility for users. This system is created to facilitate visitors' exploration and learning experiences, focusing on the extensive collection of Himmapan animals. These creatures, found in the Himmapan forests of Thailand, represent an encyclopedic array of both tangible and intangible cultural heritage.

A significant technological component of the Meta Quest is the Oculus Insight, developed by Meta Inc. This feature holds particular importance for our research as it enables users to navigate through different locations within the visual museum. Notably, Oculus Insight introduces the concept of six-degree-of-freedom (6DoF) controller tracking and headset positioning for augmented reality/virtual reality (AR/VR) devices. It calculates real-time positions of the headset and controllers, seamlessly translating real-world movements into corresponding virtual positions within the VR system, creating a truly immersive museum experience. Furthermore, by incorporating sensor inputs from the headset and controllers, our system can enhance precision and accuracy in positional tracking, further enhancing the overall quality of the user's experience.

#### 4.1.2 Digital blueprint design

**Fig. 2:**
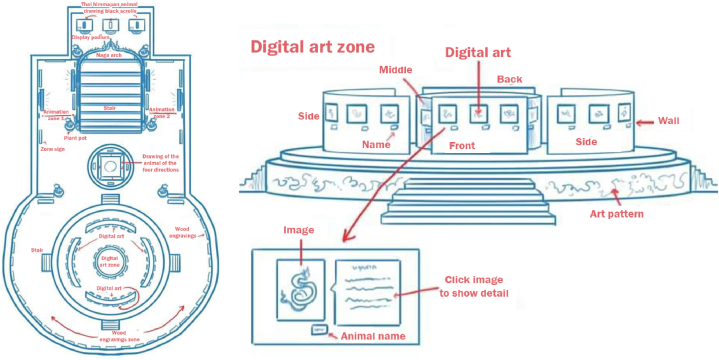
Digital blueprint design of HimmapanVR.

In our pursuit of creating a fully immersive visitor experience at the visual museum, we collaborated with the artist and designer responsible for crafting digital representations of Himmapan animals. This collaboration was in partnership with the Roitawarabarn Baandhawalai Museum. To provide the designer with a concise overview of the visual museum, we conveyed that it showcases over 300 pieces of art and crafts from the Himmapan Forest, serving as a testament to Thailand's rich cultural heritage. These pieces include digital animations, paintings, digital art books, and wall sculptures, as illustrated in Fig. 2.

To initiate the next phase, we enlisted the services of a 3D modeler tasked with creating three-dimensional models of the visual museum in collaboration with Maya Studio software. The visual museum is divided into four distinct zones, each meticulously designed to offer a comprehensive visual representation of different heritage objects.

In "Zone A," when a user enters, the scene materializes in the center of the physical room. This zone houses an exquisite collection of Himmapan animal paintings contributed by various artists from Thailand. These paintings adorn the central walls of the room.

"Zone B″ features wall sculptures that authentically depict the appearances of various Himmapan animals, sourced from actual sculptures found at the Roitawarabarn Baandhawalai Museum.

Adjacent to the ladder leading to the throne, "Zone C″ offers an interactive animation experience centered around Himmapan animals, providing an engaging and educational encounter.

**Fig. 3:**
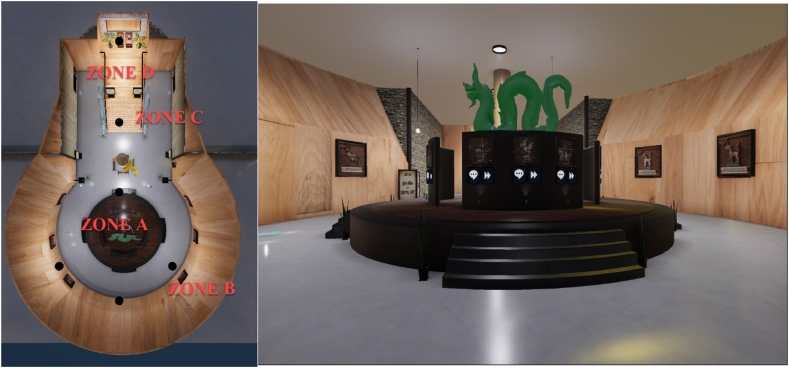
An example of distinct zones and a first-person camera view from the Visual Museum of HimmapanVR.

Lastly, at the topmost part of the room, "Zone D″ showcases a majestic throne alongside the "Thai Dum book" from the reign of King Rama III. This zone is home to a complete collection of seventy-seven types of creatures found in the Himmapan forests, further emphasizing the rich cultural significance of these mythical beings. Fig. 3 provides a visual representation of the four distinct zones, including a first-person camera view, from the Visual Museum of HimmapanVR.

#### 4.1.3 Application design and user accessibility

**Fig. 4:**
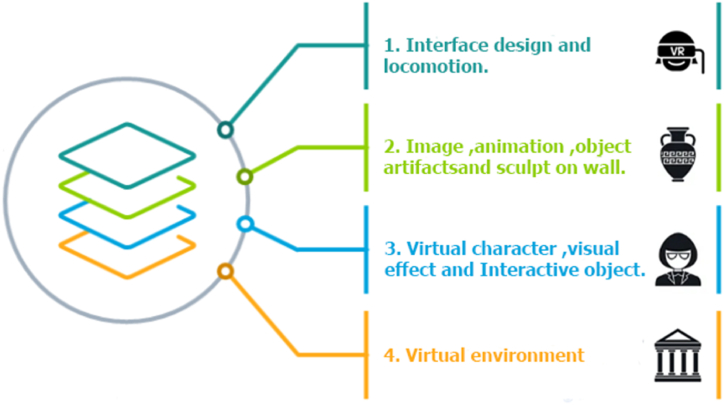
The design structure of the HimmapanVR Museum.

The focal point of our application design and user accessibility efforts is to ensure a user-friendly design that enables visitors to comprehend, navigate, and interact seamlessly with our visual reality equipment within the museum. To eliminate confusion, we have taken a meticulous approach to the fundamental design aspects, specifically focusing on the number of visualizations and interactive triggers integrated into the system. This design approach serves as a means of communication between the development team and the users, and it is depicted in Fig. 4.

The first layer of this approach pertains to the visitor interface design and the virtual reality locomotion techniques employed for visitor interaction controls.

In the second layer, we showcase various types of digital collections related to Himmapan animals. These collections do not require direct interaction but are presented to visitors in multiple forms, including images, animations, object artifacts, and wall sculptures.

Moving on to the third layer, our objective is to engage visitors through interactive elements and media. This includes virtual characters that interact with visitors, immersive visual effects, interactive objects, and the Throne of Thai Dum book. These elements are designed to provide visitors with an experience that closely mimics real-life interactions.

The fourth and final layer encompasses the entire virtual environment of the museum, detailing the room layout, floor structure, ladder placement, and the overall architectural aspects of the museum. This layer serves to provide visitors with a complete and immersive experience within the digital museum setting.

### 4.2 Digitization of cultural heritage objects

In this section, we provide an overview of the digitization process applied to each cultural heritage object within the virtual museum, which comprises four main types and fifteen sub-types of Himmapan Animals, as displayed in the museum. Given the diverse range of objects, we utilize various digital technology methods, such as image scanning, photogrammetry, and 3D modeling, to capture and preserve each cultural heritage item. The processes are outlined below.

#### 4.2.1 Thai Dum book and paint art

In our efforts to comprehensively showcase the diverse range of creatures within the visual museum, our primary goal is to digitally archive the "Thai Dum book" from the reign of King Rama III, which meticulously documents seventy-seven unique Himmapan creatures. Additionally, we aim to capture the essence of various paintings adorning the Roitawarabarn Baandhawalai Museum. To achieve this, we have adopted a digitization method in alignment with established standards [16]. This digitization process unfolds in three key steps. Firstly, we meticulously prepare the physical objects by removing debris and ensuring they are in optimal condition for digitization. Subsequently, we employ the Fujitsu Image Scanner SV600, a specialized piece of equipment capable of scanning two-dimensional data, thereby transforming height and width measurements into digital format. Finally, we enrich each digital object with essential metadata, facilitating information visualization and efficient categorization of the diverse groupings of creatures depicted. This rigorous approach ensures the accurate preservation and accessibility of our cultural heritage objects, enabling visitors to explore and appreciate the profound history and artistic intricacies of Himmapan creatures and the associated paintings.

#### 4.2.2 Archaeological objects and wall sculptures

**Fig. 5:**
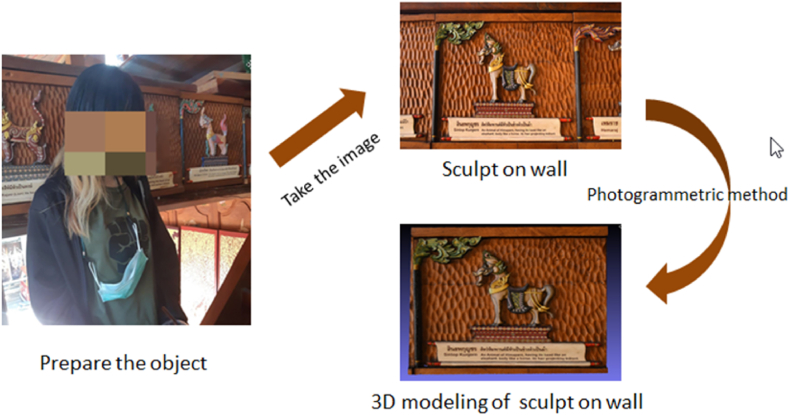
The process of generating a 3D sculpture on the wall.

In documenting the diverse heritage objects related to Himmapan animals at the Roitawarabarn Baandhawalai Museum, which includes archaeological artifacts and wall sculptures, we aimed to digitally encapsulate these items within our virtual museum through 3D modeling. By employing the photogrammetric method, as outlined in Ref. [17], utilizing digital image capture and computational techniques, we crafted 3D digital representations of these unique objects. The process began with the meticulous cleaning of each item, followed by capturing multiple images from various angles using an iPhone 12 Pro Max. This approach facilitated the accurate computation of the objects' geometries. Specifically, our photography strategy involved executing multiple passes from three distinct levels and incorporating additional close-up shots of areas with intricate details or complex shapes to ensure comprehensive coverage. The iPhone was manually maneuvered to achieve varied angular perspectives, vital for capturing detailed geometries, with an average of 100 images taken per object at a resolution of 4032 × 3024 pixels. These images were captured with the smartphone's built-in image enhancement filters deactivated to preserve the original textures and details. Utilizing Agisoft Metashape software, we processed these images to compute the 3D models. The detailed methodology, image capture strategy, and the computational processing employed are thoroughly documented and illustrated in Fig. 5, aiming to provide a clearer insight into the technical specificities and procedural nuances of our photogrammetric process in accurately digitalizing the heritage objects.

#### 4.2.3 Digital paint art and animation

**Fig. 6:**
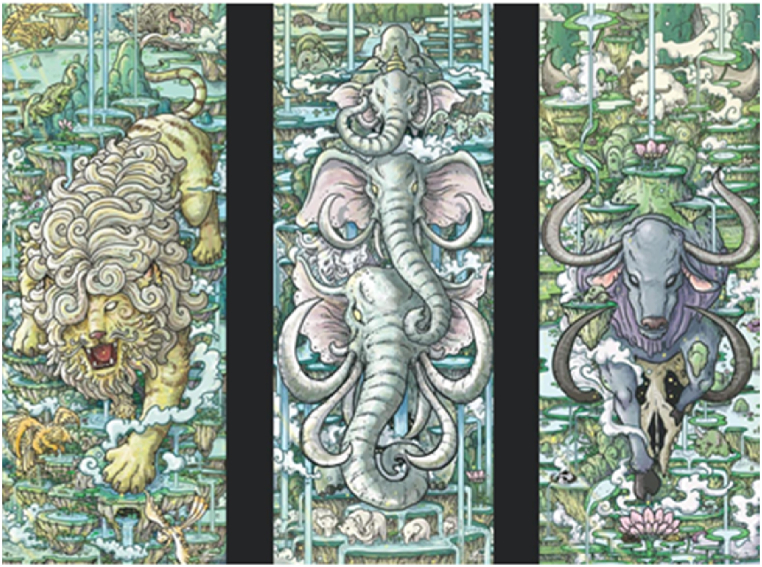
The digital artworks featuring Lion-based, Elephant-based, and Cattle-based creatures from the Himmapan Animal collection in the virtual museum.

In contrast to other cultural heritage objects in the visual museum, digital paintings and animations were initially created in digital formats, eliminating the need for a digitization process. Instead, we acquired these digital artworks through a crowdsourcing method [18]. We engaged with the community through social media campaigns, inviting artists to contribute their works depicting Himmapan animals. It is worth emphasizing that explicit permission was obtained from each artist to share and exhibit their creations in our museum. Fig. 6 showcases the digital artworks featuring Lion-based, Elephant-based, and Cattle-based creatures from the Himmapan Animal collection in the virtual museum.

### 4.3 Development and creation of digital structures

**Fig. 7:**
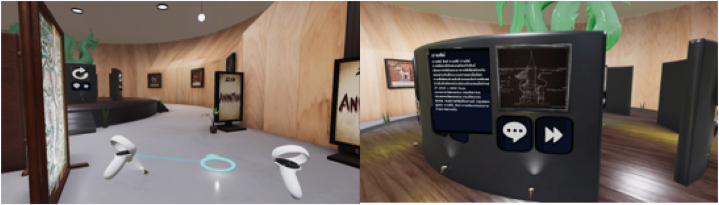
Participants utilizing the Meta Quest 2 to navigate by Point & Teleport (left) and to explore the exhibit(right) within HimmapanVR.

The goal of creating the digital structure is to design the software framework following the digital blueprint design, which consists of four systems for the visual museum. The first system includes interface design and the locomotion system, both of which aim to establish communication with the user through motion controllers and the user interface, facilitating interactions with objects in the museum. In this approach, we have chosen the "Point & Teleport" method as the most suitable locomotion technique [20]. This technique allows users to actively engage with and explore the collection. In a brief technical explanation of this locomotion method, users can hold down a button on the controller to project a virtual ray-casting line onto the ground. Releasing the button teleports the user to their selected location, facilitating exploration of the exhibit as illustrated in Fig. 7. The second system is dedicated to organizing the various digital file types of the digital collection, creating a coherent art gallery featuring Himmapan animals. Given the diversity of file formats to manage, including images, animations, sounds, and 3D objects, we have developed a robust file management system that assigns metadata to each object for efficient categorization and visualization.

**Fig. 8:**
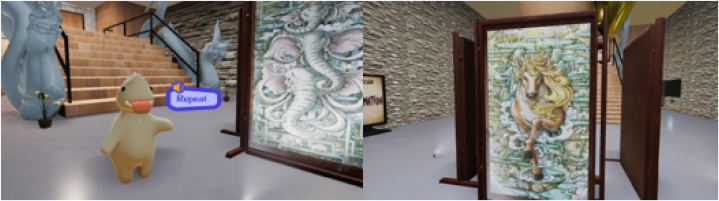
Virtual characters of himmapan marshmello to support the visitors.

The objective of the third system is to immerse users in the experience of observing the antiques. Virtual characters are designed to appear in the center of the museum, where users can press a button to access audio descriptions for each antique, as depicted in Fig. 8. Additionally, this system is responsible for representing animated and interactive objects. Finally, the last system implements the model of the Himmapan animals' museum, following the digital blueprint. It creates the 3D digital model using Autodesk Maya 2022 and imports it into the Unity game engine to display the graphics. It also defines the boundaries of the area to limit users' movement and observation within the virtual museum.

**Fig. 9:**
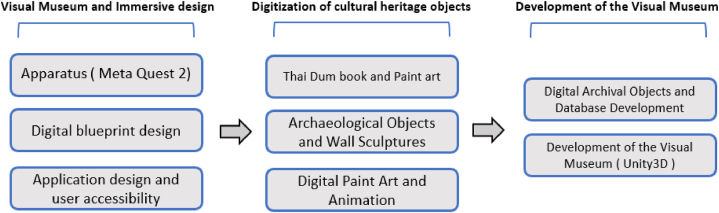
Overview of development process of HimmapanVR.

#### 4.3.1 Digital archival objects and database development

In particular, we have implemented a specifically designed database for the digital archival objects within the visual museum. This database serves as a repository for all the various objects related to Himmapan animals. To accomplish this, we structured the database in accordance with the data standards for describing works of visual culture known as VRA Core metadata [19]. These standards were created by the Visual Resources Association in collaboration with the MARC Standards Office of the Library of Congress (LC). We chose to develop the database using SQLite Database, ensuring efficient organization and management of our digital archival objects.

#### 4.3.2 Development of the visual museum

In the development phase, we opted for Unity 2021.1a as the core graphic visualization software for the visual museum. This choice was made because our team was well-versed in using the Unity engine, and it offers compatibility across multiple platforms, making it highly user-friendly for both experts and beginners. Considering the diverse range of virtual reality headsets available, we decided to develop the visual museum specifically for the Meta Quest 2 headset, as it is one of the most popular virtual reality headsets in the market and offers cost advantages compared to other options. All the content related to Himmapan animals was seamlessly integrated into the Unity3D game engine, which was then compiled to create the virtual reality application for the Meta Quest 2 headset. Throughout this process, we collaborated closely with the Department of Digital Game at the College of Art, Media, and Technology, Chiang Mai University, benefiting from their expertise in media technology and digital game development.

## 5 Research design and hypotheses

In this study we used the research and development method. According to Ref. [23] research and development (R&D) can use as the method to develop and prototype and evaluate the effectiveness of the virtual museum. To assess the visitor experience and educational impact of our designed virtual museum of Himmapan animals, we considered the support and enhancement capabilities of virtual reality headsets. We formulated hypotheses to evaluate the user experience within the virtual museum, drawing inspiration from the concepts of Effort Expectancy (EE) and Performance Expectancy (EP) [21,22], which are commonly utilized in User Experience (UX) design and evaluation. EE is defined as "the perceived ease of use associated with the system," and it is employed to gauge the level of effort users believe is necessary to accomplish specific tasks or objectives when interacting with a virtual museum. Similarly, PE is defined as "the extent to which an individual believes that using the system will improve their job performance." This concept is used to assess user expectations regarding the performance and functionality of a visual museum, its systems, and interface [21].

### 5.1 Our hypotheses are as follows

Hypothesis 1 (H1): The implementation of the Virtual Museum of Himmapan animals has a positive impact on Effort Expectancy (EE). Users will perceive the system as user-friendly and easy to use.

Hypothesis 2 (H2): The implementation of the Virtual Museum of Himmapan animals positively influences Performance Expectancy (EP). Users anticipate that the system will enhance their job performance and overall experience when interacting with the visual museum.

Hypothesis 3 (H3): The virtual Museum of Himmapan can effectively visualize the realism of the archaeological objects related to the Himmapan cultural heritage.

Hypotheses H1 and H2 are pivotal in our research as they form the cornerstone for assessing the user experience and educational impact of the virtual Museum of Himmapan animals. These hypotheses are crucial as they directly address the user-centric aspects of our virtual museum, aligning with the fundamental goal of providing an engaging and educational experience. Furthermore, H3 underscores the significance of accurately visualizing archaeological objects related to Himmapan cultural heritage, emphasizing the need for fidelity and authenticity in digital representation as a critical factor in the preservation and presentation of cultural artifacts within a virtual museum context.

The testing of Hypotheses H1, H2, and H3 involves evaluating the Virtual Museum of Himmapan animals' user experience and educational benefits. For H1, participants' ease of use is assessed via questionnaires focusing on their VR familiarity, navigation confidence, and overall user-friendliness. H2's evaluation targets the museum's impact on enhancing knowledge of Himmapan cultural heritage, measured through participants' engagement and learning experiences. H3 examines the museum's effectiveness in realistically visualizing archaeological objects, with feedback gathered on the authenticity and impact of these representations on cultural appreciation. Participants explore the museum using the Meta Quest 2 VR headset and subsequently provide feedback through questionnaires, enabling a concise evaluation of the virtual museum's usability, educational impact, and authenticity portrayal.

### 5.2 Participants

We recruited 30 participants for data collection and feedback from visitors of the Roitawarabarn Baandhawalai Museum through face-to-face invitations. The participant demographics consisted of 56.66 % females and 43.33 % males, with an average age of 32 years, ranging from 25 to 37. To encourage their active engagement, we provided a financial compensation of 100 baht (approximately 3 USD). This compensation was intended to recognize the time and effort invested by participants and to incentivize them to provide high-quality data via the set of questionnaires.

### 5.3 Instrument and measurement

Our testing approach for the HimmapanVR project entailed a comprehensive evaluation strategy that incorporated quantitative methodologies. To assess user experience (UX) dimensions, such as Effort Expectancy (EE) and Performance Expectancy (PE), and to gain valuable insights into both usability and the educational impact of the virtual museum, we administered three sets of structured questionnaires designed to collect feedback from participants.

**Effort Expectancy Questionnaire:** This questionnaire set was devised to validate the first hypothesis, emphasizing the user-friendliness of virtual reality headsets. The assessment encompassed factors such as navigation, interaction, and ease of use within the museum environment which was employed and adapt from Ref. [21]. Detailed information regarding the first questionnaire set can be found in Table 3.

**Table 3:** First set of questionnaires for Hypothesis 1 (H1).

No.	Question.	Mean (SD)
H1.Q1	How familiar are you with Virtual Reality?	4.06 (0.82)
H1.Q2	How confident are you using and navigating the virtual Museum through a Virtual Reality headset?	3.96 (0.61)
H1.Q3	What level of interaction have you experienced using Virtual Reality as an educational tool for cultural heritage?	4.20 (0.62)
H1.Q4	Did you require any assistance while using the Virtual Reality headset?	3.70 (0.59)
H1.Q5	Overall, how easy was it to use the Virtual Reality headset to observe the virtual museum?	3.86 (0.79)

**Performance Expectancy Questionnaire:** The second set of questionnaires was aimed at evaluating users' engagement with Virtual Reality functionalities and their experience within the 3D environment of the visual museum [21,22]. This set sought to measure the impact of the virtual museum on participants' learning about cultural heritage and their perceived usefulness of Himmapan animals in real-life contexts. Detailed information about the second questionnaire set is available in Table 4.

**Table 4:** Second set of questionnaires for Hypothesis 2 (H2).

No.	Question.	Mean (SD)
H2.Q1	How much knowledge do you have about Himmapan animals as a cultural heritage in Thailand?	3.56 (0.85)
H2.Q2	What has been your level of recognition of the Himmapan animals as cultural heritage in real life?	3.86 (0.69)
H2.Q3	Did you learn interesting information during your interaction with the virtual reality application?	4.16 (0.59)
H2.Q4	Did the virtual reality application provide you with a unique experience regarding Himmapan animals?	4.23 (0.67)
H2.Q5	Overall, how appropriate did you find the Virtual Reality headset for gaining knowledge about Himmapan animals?	4.03 (0.76)

**Realism of the Archaeological Objects Questionnaire:** In order to address the third hypothesis concerning the accurate visualization of archaeological objects related to Himmapan cultural heritage, a third set of questionnaires was administered. This questionnaire set was designed to assess participants' perceptions of the realism and authenticity of the archaeological objects presented within the virtual museum. Further details about the third questionnaire set can be found in Table 5.

**Table 5:** Third set of questionnaires for Hypothesis 3 (H3).

No.	Question.	Mean (SD)
H3.Q1	To what extent did the realism of the archaeological objects encountered in the virtual museum impact your perception?	3.81(0.55)
H3.Q2	How much did the virtual museum's portrayal of archaeological objects contribute to your understanding and appreciation of Himmapan cultural heritage?	3.85 (0.61)
H3.Q3	How accurately do you believe the virtual museum represented the archaeological objects related to Himmapan cultural heritage?	4.01 (0.69)
H3.Q4	Overall, how effective do you believe the virtual museum was in preserving and presenting Himmapan cultural artifacts?	4.22 (0.45)

### 5.4 Procedure

The researcher initiated the session by providing a detailed description of the research purpose and process. Subsequently, participants were presented with an electronic consent form and were requested to provide their consent by signing it. Following the consent process, participants were introduced to the virtual reality device, specifically the Meta Quest 2 headset, and were instructed on how to properly wear and adjust the headset straps for optimal comfort and usability. Each participant was allotted 30 min to explore the Virtual Museum of Himmapan animals. After concluding their virtual museum visit, participants were administered a series of questionnaires meticulously crafted to assess and validate our hypotheses. These questionnaires featured 5-point Likert scale items.

## 6 Result

**Fig. 10:**
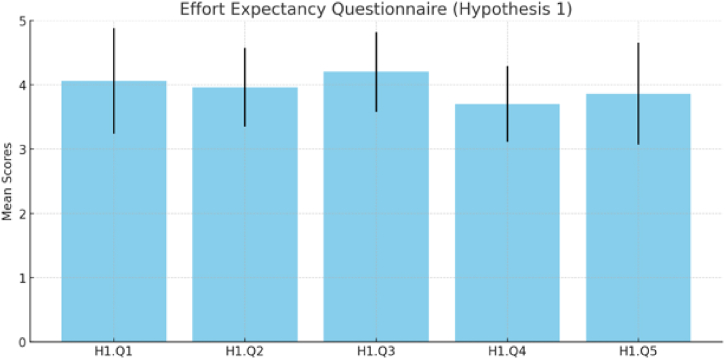
Result of expectancy questionnaire (Hypothesis 1).

The responses to Hypothesis 1 (H1) regarding Effort Expectancy (EE) are presented in Fig. 10 and Table 3. Notably, the results for the initial questions (H1.Q1 and H1.Q2) indicate a high level of familiarity with and confidence in navigating the virtual reality (VR) environment (Mean = 4.06, Mean = 3.96). Furthermore, participants express a positive view of VR's effectiveness as an educational tool for cultural heritage material (H1.Q3, Mean = 4.20). However, it is noteworthy that setting up the VR headset posed challenges for new participants in the virtual reality experience, necessitating support and assistance (H1.Q4, Mean = 3.70). Consequently, participants' overall impression of our system with the Virtual Reality headset, as assessed in H1.Q5, indicates some difficulties in observing the virtual museum, primarily attributed to headset setup issues (Mean = 3.86).

**Fig. 11:**
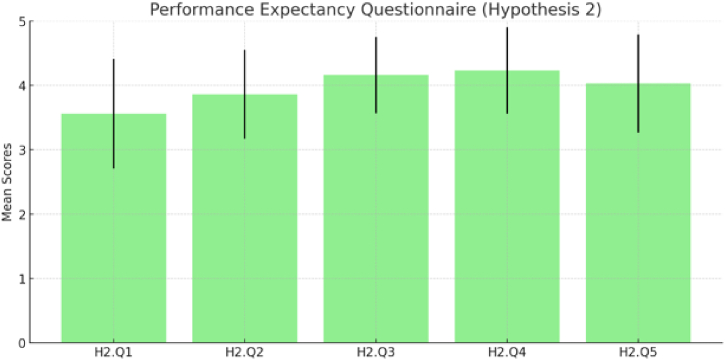
Result of performance expectancy questionnaire (Hypothesis 2).

Fig. 11 and Table 4 provides a succinct summary of participant responses in the context of Hypothesis 2 (H2) regarding EP and the influence of knowledge about Himmapan animals. This second set of questionnaires aimed to assess participants' knowledge and perceptions of Himmapan animals as a cultural heritage in Thailand. The mean values and standard deviations offer valuable insights into participant opinions. Notably, participants demonstrated a moderate level of prior knowledge (H2.Q1: Mean = 3.56, SD = 0.85) and recognition of Himmapan animals in real life (H2.Q2: Mean = 3.86, SD = 0.69). Moreover, participants indicated that the virtual museum significantly contributed to their understanding and appreciation of Himmapan cultural heritage (H2.Q3: Mean = 4.16, SD = 0.59). They also perceived the virtual reality application as providing a unique and enriching experience (H2.Q4: Mean = 4.23, SD = 0.67). Overall, participants found the Virtual Reality headset highly suitable for acquiring knowledge about Himmapan animals (H2.Q5: Mean = 4.03, SD = 0.76).

**Fig. 12:**
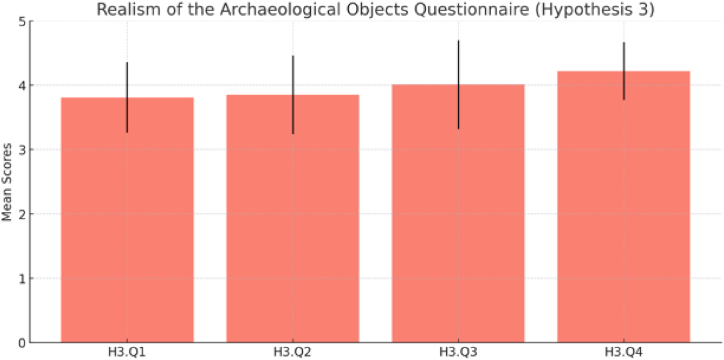
Result of realism of the archaeological objects questionnaire (Hypothesis 3).

Fig. 12 and Table 5 offers an overview of participant responses concerning Hypothesis 3 (H3). This third set of questionnaires aimed to assess participants' perceptions of the realism and authenticity of archaeological objects within the virtual museum. On average, participants provided positive feedback, indicating a moderate level of perceived realism for the archaeological objects (H3.Q1: Mean = 3.81, SD = 0.55) and a similar perception of accuracy in representing Himmapan cultural heritage (H3.Q2: Mean = 3.85, SD = 0.61). Moreover, participants highlighted the significant contribution of the virtual museum's portrayal of archaeological objects to their understanding and appreciation of Himmapan cultural heritage (H3.Q3: Mean = 4.01, SD = 0.69). Impressively, participants found the representation highly effective in preserving and presenting Himmapan cultural artifacts (H3.Q4: Mean = 4.22, SD = 0.45).

### 6.1 Summary of the result of hypotheses

The summary of the hypotheses results combines the findings from three distinct investigations into the effectiveness of a virtual reality (VR) environment for cultural heritage education. The first hypothesis (H1) highlights the participants' high level of familiarity and confidence with navigating the VR environment and their positive perception of VR as a tool for cultural heritage education, while also pointing out the challenges in setting up the VR headset and difficulties in observing the virtual museum, which suggests the need for addressing these usability issues to enhance the user experience. To overcome such challenges, for example, Pure Land projects uses a combination of high-resolution photography and 3D architectural models with immersive display technologies, allowing users to navigate and interact with the virtual environment intuitively. Adopting similar strategies can help address challenges related to setting up VR headsets and observing virtual museum exhibits, making the VR environment more accessible and enjoyable for users [34]. The second hypothesis (H2) illustrates the virtual museum's success in enhancing participants' understanding and appreciation of the Himmapan cultural heritage, marked by positive feedback and an improvement in the recognition and prior knowledge of Himmapan animals. The third hypothesis (H3) focuses on the virtual museum's ability to present archaeological objects with a sense of realism and authenticity, which has significantly contributed to the participants' appreciation of the Himmapan cultural heritage. Despite the overall effectiveness, the variability in responses hints at the necessity for further improvement in realism for a more universally positive experience among participants. Drawing on the Pure Land projects' findings, applying high-resolution digital data to create accurate and detailed representations of Himmapan animals and other archaeological objects can enhance the sense of realism in the virtual museum, contributing to a more universally positive experience among participants [34].

## 7 Discussion

Virtual museums offer significant benefits in terms of preservation and education through the use of three-dimensional content technology [26]. Visitors can explore virtual museums at their own pace, which is often more convenient compared to physical locations [27]. Moreover, artworks and object artifacts are visualized with interactivity, providing a more immersive experience [28,29]. In our approach, we have employed full virtual reality technology to reconstruct virtual museums dedicated to Himmapan animals. This initiative aims to conserve and preserve the knowledge of Himmapan animals, contributing to the cultural heritage of Thailand.

The results from our study highlight the potential of VR as a powerful tool for cultural preservation and education. Participants reported high levels of engagement and increased knowledge about the Himmapan animals, reflecting the effectiveness of the virtual museum in enhancing the dissemination of cultural heritage. However, our findings also underscored the challenges related to usability and the need for further enhancements to fully realize the educational and experiential possibilities of VR. The discussion section of our paper now delves deeper into these aspects, considering the implications of our findings for the development of future VR applications in cultural preservation. We also reflect on the limitations of our study, including the choice of scales for measuring UX.

### 7.1 Challenge and urgency of VR development for cultural preservation

**Fig. 13:**
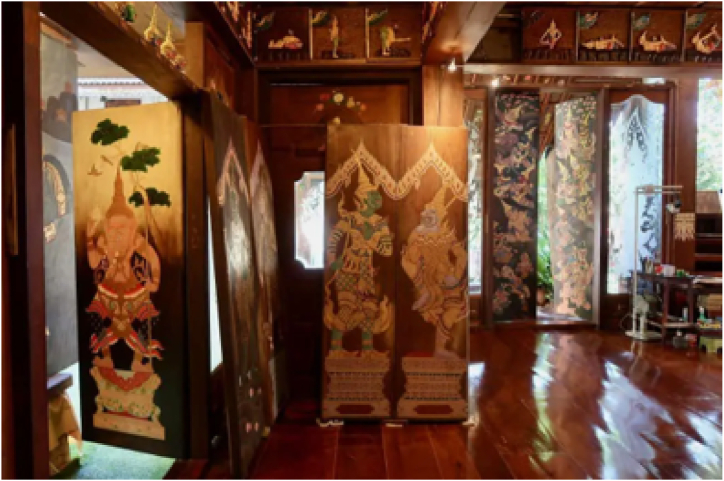
Roitawarabarn Baandhawalai museum.

The urgency of developing Virtual Reality (VR) applications for cultural preservation is increasingly recognized amidst rapid technological advancements and the evolving needs of society. This urgency becomes particularly pronounced in the preservation of intangible cultural heritage, such as the mythical narratives surrounding the Himmapan creatures, which are at risk of being lost or diminished in the absence of innovative preservation strategies. The HimmapanVR project was initiated in response to this pressing need, aiming to leverage VR technology to safeguard and revitalize the rich tapestry of Thai cultural heritage for future generations. Ensuring the accurate representation and seamless adaptation of this knowledge into digital formats is of paramount importance to maintain the authenticity of the exhibits. To accomplish this task, we heavily relied on the 'Thai Dum book' from the Fine Arts Department, Ministry of Culture, Thailand, as our primary reference source. This comprehensive resource significantly aided us in categorizing and archiving the Himmapan animals. Moreover, we collected artefact objects related to Himmapan animals from the Roitawarabarn Baandhawalai Museum, which houses numerous art objects rooted in the mythological elements resulting from a complex fusion of Hindu, Buddhist, Chinese, and Thai cultures, as shown in Fig. 13.

Digitizing cultural heritage objects and achieving interactivity and immersion in the context of mythical creatures is a complex endeavor that demands innovative storytelling and the integration of technology to effectively engage visitors. The digitization process involved employing advanced 3D survey techniques, including digital photogrammetry. Capturing objects in various conditions, such as differing light conditions and from close distances, introduced additional challenges due to the limited depth of field. This necessitated skilled photography to obtain sharp and well-contrasted images. It's worth noting that environmental conditions, particularly lighting, had a significant impact on passive shooting techniques [31]."

### 7.2 The participants in the virtual museum

Based on the results of the three hypotheses, it is evident that HimmapanVR significantly enhances participants' familiarity and confidence in navigating the virtual reality environment (H1) and effectively boosts their knowledge of Himmapan animal cultural heritage (H2). Furthermore, it successfully conveys a profound sense of realism and authenticity in the representation of archaeological objects (H3). To further enhance participants' experiences, we recommend the adoption of three key technologies. Firstly, embracing a fully immersive virtual reality setup can provide an unparalleled sense of presence, addressing the limitations often associated with virtual museums that lack virtual reality support [30]. Secondly, we propose the use of high-quality photogrammetry software [32,33] to meticulously create realistic and detailed representations of archaeological objects. Finally, regarding challenges encountered during the setup of VR headsets for new participants, and difficulties in navigating the virtual museum which suggest potential usability issues a possible solution could be to simplify the VR headset setup for new users. This could be achieved by developing a more intuitive, step-by-step onboarding guide. Such a guide would include visual instructions and interactive tutorials designed to familiarize users with the equipment before they commence their virtual museum tour. This approach will enable participants to engage more interactively with the objects, ultimately heightening the overall sense of presence, which is of paramount importance in the ongoing development of the virtual museum.

### 7.3 Limitation of study

The HimmapanVR project has demonstrated potential in enriching the dissemination and appreciation of Thai cultural heritage through an immersive virtual reality experience. Recognizing the limitations identified in our study is crucial, particularly in assessing the educational impact and user experience (UX) of our VR application. Our research adopted the Effort Expectancy/Performance Expectancy scale, a choice informed by its reliability in measuring the usability aspects of technological applications. This scale was chosen for its straightforward approach to quantifying user interactions and the perceived ease and effectiveness of using the VR technology. However, we acknowledge that this scale may not sufficiently capture the entire spectrum of user experiences, especially those related to the hedonic (pleasure-related) and eudaimonic (meaning-related) dimensions [33] that virtual reality uniquely offers. This limitation is significant, given VR's capability to provide deeply immersive and emotionally engaging experiences that extend beyond mere usability.

While the selected questionnaire provided valuable insights into the functional aspects of interacting with the HimmapanVR, it might have overlooked the nuanced and transformative educational experiences facilitated by the application. The complexity and depth of learning about cultural heritage through virtual reality likely surpass the metrics measured by our chosen scale, potentially neglecting the profound and multifaceted educational impacts VR can have. Moreover, our study encountered challenges in fully conveying the rich narratives and cultural significance of Himmapan mythology within the virtual reality framework. Although VR offers innovative avenues for cultural education, the digitization process may not completely capture the complex stories and cultural essence of Himmapan creatures, thus affecting the depth of educational content delivered.

Additionally, usability issues identified by participants, such as navigation difficulties and the need for assistance with the VR headset, indicate that technical barriers may detract from the immersive learning experience and accessibility of the application, particularly for individuals new to VR technology. The study's limited sample size and the demographic diversity of participants further constrain the generalizability of our findings regarding the application's effectiveness in cultural education.

## 8 Conclusion and future research

In conclusion, we have successfully developed the first Virtual Museum dedicated to the cultural heritage of Himmapan animals, a digital repository that showcases the rich traditions and mythological significance of these creatures in Thai culture. They are prominently featured in various art forms, literature, traditional performances, and architectural designs. This museum serves as a vital contributor to the preservation and accessibility of these cultural treasures and also plays a significant role as an educational resource. Our user survey results highlight its superiority over traditional archives, underscoring its utility and user-friendliness in promoting Thai cultural heritage.

Future research in this field should focus on enhancing the user experience, especially for newcomers to virtual reality, by improving headset setup procedures and incorporating advanced technologies. Additionally, exploring the integration of Mixed Reality (MR) to augment the virtual reality framework could offer users a more immersive experience. Investigating the long-term cultural and educational impacts of the virtual museum across diverse user demographics will be essential for evaluating its sustained relevance and effectiveness. Moreover, while this study primarily focused on user perceptions and experiences, the broader cultural and societal impacts of the virtual museum warrant further exploration. Finally, variations in participant expectations and preferences regarding virtual artefact representation suggest the need for more in-depth investigations and content tailoring to enhance overall user satisfaction.

## Data availability statement

The data associated with this study have not been deposited into a publicly available repository. The data associated with this study will be made available on request.

## CRediT authorship contribution statement

**Suepphong Chernbumroong:** Writing – original draft. **Perasuk Worragin:** Software. **Natchaya Wongwan:** Writing – original draft. **Kannikar Intawong:** Software. **Pipitton Homla:** Investigation, Formal analysis, Data curation. **Kitti Puritat:** Writing – original draft, Methodology, Conceptualization.

## Declaration of competing interest

The authors declare that they have no known competing financial interests or personal relationships that could have appeared to influence the work reported in this paper.
