# Supplementary material for: Digitization of myth: The HimmapanVR Project's role in cultural preservation
Source: Heliyon. 2024 Apr 20;10(9):e30052. doi: 10.1016/j.heliyon.2024.e30052 (PMC11061741; doi:10.1016/j.heliyon.2024.e30052)
Supplement: Multimedia component 1 [file mmc1.docx]

Appendix

1.Effort Expectancy (EE) Questionnaire (five-point Likert scale ranging from strongly disagree to strongly agree)

| No | Question |
| --- | --- |
| 1 | How familiar are you with Virtual Reality? |
| 2 | How confident are you using and navigating the virtual Museum through a Virtual Reality headset? |
| 3 | What level of interaction have you experienced using Virtual Reality as an educational tool for cultural heritage? |
| 4 | Did you require any assistance while using the Virtual Reality headset? |
| 5 | Overall, how easy was it to use the Virtual Reality headset to observe the virtual museum? |

2.Performance Expectancy (PE) Questionnaire (five-point Likert scale ranging from strongly disagree to strongly agree)

| No. | Question. |
| --- | --- |
| 1 | How much knowledge do you have about Himmapan animals as a cultural heritage in Thailand? |
| 2 | What has been your level of recognition of the Himmapan animals as cultural heritage in real life? |
| 3 | Did you learn interesting information during your interaction with the virtual reality application? |
| 4 | Did the virtual reality application provide you with a unique experience regarding Himmapan animals? |
| 5 | Overall, how appropriate did you find the Virtual Reality headset for gaining knowledge about Himmapan animals? |

3. Realism of the Archaeological Objects Questionnaire (five-point Likert scale ranging from strongly disagree to strongly agree)

| No. | Question. |
| --- | --- |
| 1 | To what extent did the realism of the archaeological objects encountered in the virtual museum impact your perception? |
| 2 | How much did the virtual museum's portrayal of archaeological objects contribute to your understanding and appreciation of Himmapan cultural heritage? |
| 3 | How accurately do you believe the virtual museum represented the archaeological objects related to Himmapan cultural heritage? |
| 4 | Overall, how effective do you believe the virtual museum was in preserving and presenting Himmapan cultural artifacts? |
